# Supplementary material for: Intraamniotic Zika virus inoculation of pregnant rhesus macaques produces fetal neurologic disease
Source: Nat Commun. 2018 Jun 20;9:2414. doi: 10.1038/s41467-018-04777-6 (PMC6010452; doi:10.1038/s41467-018-04777-6)
Supplement: Supplementary file 1 — Supplementary Information [file 41467_2018_4777_MOESM1_ESM.pdf]

Intraamniotic Zika Virus Inoculation of Pregnant Rhesus Macaques Produces Fetal Neurologic Disease

Coffey et al.

Supplementary Table 1

System	Tissue	GD41 fetus	GD50 fetus	GD64 neonate	GD90 fetus		GD41 mother	GD50 mother	GD64 mother	GD90 mother	animal
		41	50	64	90		41	50	64	90	inoculation (GD)
		48	155	151	155		48	155	151	155	necropsy (GD)
		7	105	87	65		7	105	87	65	necropsy dpi
Lymphoid	Tonsil	nc	WNL	WNL	nc		Mild LH	Moderate LH	ne	ne	
	Retropharyngeal LN	nc	nc	nc	nc		nc	Mild LH	Moderate LH	Mild LH	
	Submandibular LN	nc	WNL	EMH	EMH		ne	ne	ne	ne	
	Cervical LN	nc	nc	nc	nc		nc	WNL	Mild LH	Mild LH	
	Axillary LN	nc	Mild edema	EMH	EMH		ne	ne	ne	ne	
	Bronchial LN	nc	nc	nc	nc		nc	nc	Moderate LH	Moderate pneumoconiosis	
	Mesenteric LN	nc	Mild LH, EMH	EMH	EMH		ne	ne	ne	ne	
	Peri-aortic LN	nc	nc	nc	nc		nc	WNL	WNL	WNL	
	Inguinal LN	nc	Moderate edema	EMH	EMH		ne	ne	ne	ne	
	Thymus	nc	WNL	WNL	WNL		ne	ne	ne	ne	
	Spleen	nc	Mild LH, EMH	EMH	EMH		ne	ne	ne	ne	
	Bone marrow	n/a	WNL	WNL	WNL		WNL	Insufficient sample	WNL	WNL	
Cardiopulmonary	Heart, including A/V valves	WNL	WNL	WNL	WNL		Rare myocardial lymphocytic infiltrates	WNL	WNL	WNL	
	Aortic valve	ne	ne	ne	ne		nc	ne	ne	ne	
	Aorta, thorax	nc	WNL	WNL	WNL		nc	WNL	WNL	WNL	
	Aorta, abdomen	WNL	nc	nc	nc		nc	ne	ne	ne	
	Pulmonary arteries	nc	nc	nc	nc		nc	ne	ne	ne	
	Femoral artery/vein	nc	WNL	WNL	WNL		nc	nc	nc	nc	
	Pericardium	nc	WNL	WNL	WNL		nc	ne	ne	ne	
	Lung, caudal lobe	WNL	Mild squames	Moderate macrophages, mild neutrophils; moderate BALT hyperplasia; moderate squames	Mild squames		WNL	WNL	Mild pulmonary edema	Mild pneumoconiosis	
	Bronchi	nc	WNL	WNL	WNL		nc	ne	ne	ne	
Digestive	Parotid SG	nc	WNL	WNL	WNL		ne	ne	ne	ne	
	Submandibular SG	nc	WNL	WNL	WNL		ne	ne	ne	ne	
	Oropharynx	nc	WNL	WNL	WNL		WNL	WNL	nc	WNL	
	Esophagus	WNL	nc	nc	nc		nc	nc	nc	nc	
	Stomach	nc	WNL	WNL	WNL		ne	ne	ne	ne	
	Duodenum	nc	WNL	WNL	WNL		nc	WNL	WNL	WNL	
	Jejunum	WNL	WNL	WNL	WNL		WNL	WNL	WNL	WNL	
	Ileum	nc	WNL	WNL	WNL		WNL	WNL	WNL	WNL	
	Colon	WNL	WNL	WNL	WNL		ne	ne	ne	ne	
	Liver, left lateral lobe	EMH	Glycogenosis, lipidosis, EMH	Mild lipidosis, EMH	Glycogenosis, lipidosis, EMH		Rare macrophages, neutrophils	Nodular hyperplasia, bridging fibrosis; moderate lipidosis, glycogenosis	Moderate glycogenosis	Mild lymphoplasmacytic portal hepatitis	
	Gall bladder	nc	WNL	WNL	WNL		WNL	Moderate lymphoplasmacytic cholecystitis	WNL	Mild lymphoplasmacytic cholecystitis	
	Pancreas	nc	WNL	WNL	WNL		nc	ne	ne	ne	
Integument	Adipose tissue	n/a	nc	nc	nc		nc	ne	ne	ne	
	Skin, intrascapular	WNL	WNL	Focal epidermal ulcer, fibrin, few neutrophils	WNL		nc	WNL	WNL	WNL	
	Skin, medial thigh	nc	WNL	WNL	WNL		nc	ne	ne	ne	
Musculoskeletal	Cartilage, L knee	WNL	WNL	WNL	WNL		nc	ne	ne	ne	
	Tendon/ligaments, L knee	n/a	WNL	WNL	WNL		nc	ne	ne	ne	
	Bone	WNL	WNL	WNL	WNL		nc	ne	ne	ne	
	Finger, distal 2 cm	WNL	nc	nc	nc		nc	nc	nc	nc	
	Muscle, quadriceps	WNL	WNL	WNL	WNL		nc	ne	ne	ne	
	Fascia	n/a	nc	nc	nc		nc	nc	nc	nc	
Genitourinary	Kidney, L	WNL	WNL	WNL	WNL		Mild lymphocytic infiltrates; mild medullary amyloidosis	Mild lymphocytic infiltrates	Mild lymphocytic infiltrates	Mild lymphocytic infiltrates	
	Urinary bladder	WNL	WNL	WNL	WNL		ne	ne	ne	ne	
	Ovary	n/a	WNL	n/a	WNL		WNL	WNL	WNL	WNL	
	Uterus/cervix/vagina	n/a	WNL	n/a	WNL		ne	ne	ne	ne	
	Testis	WNL	n/a	WNL	n/a		n/a	n/a	n/a	n/a	
	Seminal vesicles/prostate	n/c	n/a	WNL	n/a		n/a	n/a	n/a	n/a	
Neuroendocrine	Brain	WNL	Loss of ependymal cells; periventricular gliosis; decreased neuronal rests; loss of cortical layering	Loss of ependymal cells; periventricular foci of mineral, gliosis; decreased neuronal rests	Loss of ependymal cells, periventricular gliosis; decreased neuronal rests		ne	ne	ne	ne	
	Spinal cord	WNL	WNL	WNL	WNL		ne	ne	ne	ne	
	Dura Mater	n/a	WNL	WNL	WNL		nc	ne	ne	ne	
	Eye	WNL	WNL	WNL	WNL		nc	ne	ne	ne	
	Nerve, sciatic	nc	WNL	WNL	WNL		ne	ne	ne	ne	
	Nerve, brachial plexus	nc	WNL	WNL	WNL		ne	ne	ne	ne	
	Adrenal gland	WNL	WNL	WNL	WNL		ne	ne	ne	ne	
	Thyroid gland	WNL	WNL	WNL	WNL		ne	ne	ne	ne	
	Pituitary gland	nc	nc	nc	WNL		ne	ne	ne	ne	
Fetal membranes	Umbilical cord	WNL	WNL	Neutrophils and hemorrhage	WNL		n/a	n/a	n/a	n/a	
	Placenta	Decidual lymphocytes; mild chorionic plate neutrophils, trophoblast necrosis	Marginal infarction	Neutrophils in basal plate with bacteria; marginal infarction	Marginal infarction		n/a	n/a	n/a	n/a	
	Amniotic membranes	WNL	WNL	Neutrophils, bacteria, hemorrhage and mineral	WNL		n/a	n/a	n/a	n/a	

Gross and histologic observations in maternal and fetal and neonatal tissues from ZIKV infected rhesus macaques. *nc* is not collected, *ne* is not examined since no ZIKV RNA was detected in that tissue, *n/a* is not applicable/available, *LH* is lymphoid hyperplasia, *EMH* is extramedullary hematopoiesis, *LN* is lymph node, *WNL* is within normal limits.

Supplementary Figure 1

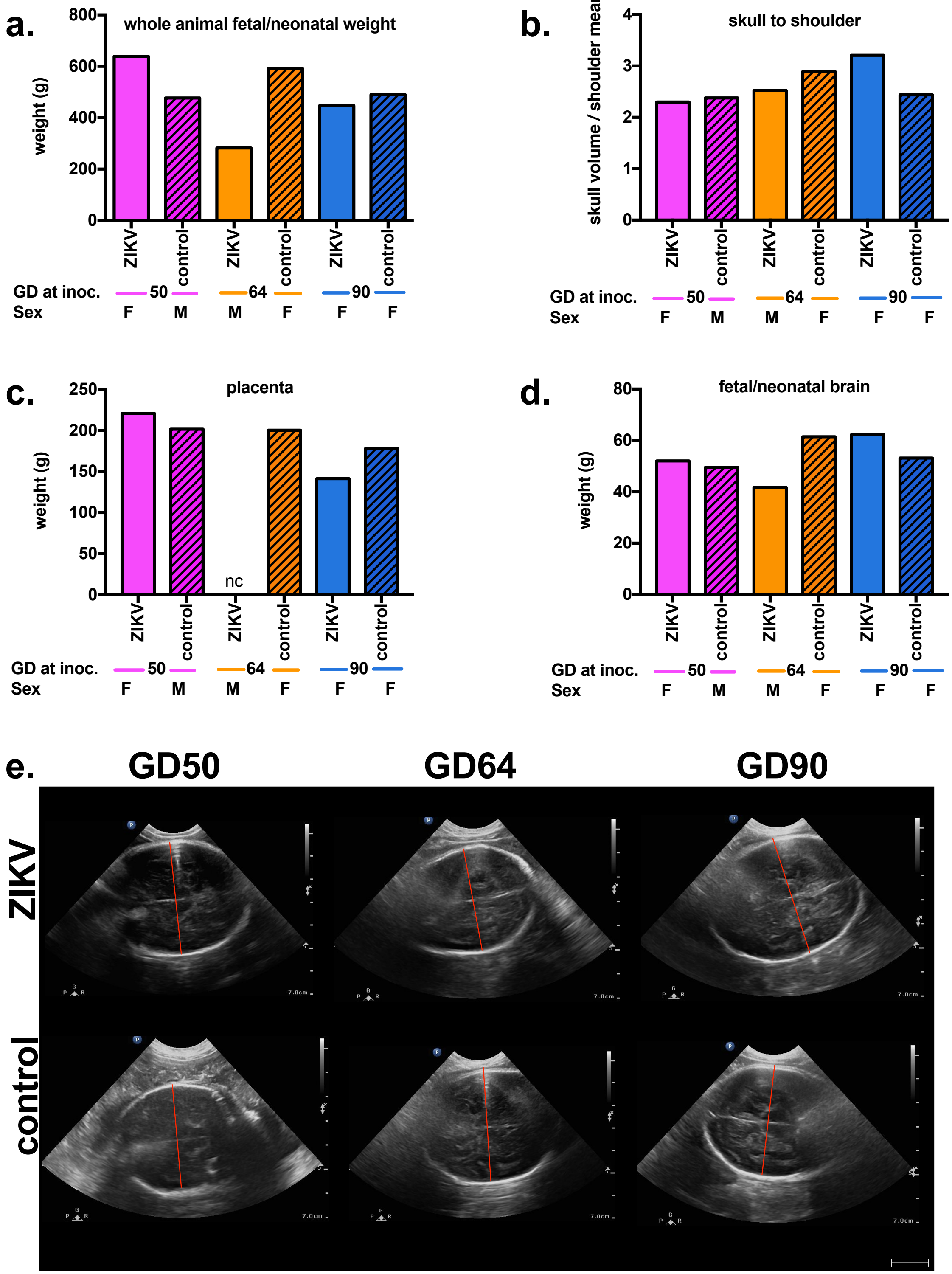

Fetal dimensions measured as **a) whole fetus weight**, **b) size**, as determined by the skull volume divided by the shoulder mean, calculated as the left and right clavicle distance, c) **placental** and d) **fetal brain** weights, in grams and **e) ultrasound**. Ultrasound images are representative and are from the last exam for each animal, with BPDs depicted as a red line. GDs were matched as best as possible: ZIKV GD50 animal US at GD150 versus the control at GD134, the ZIKV GD64 animal US at GD143 versus the control at GD141, and the ZIKV GD90 animal US at GD148 versus the control at GD139. The scale bar in e) shows 2 cm.

Supplementary Figure 2

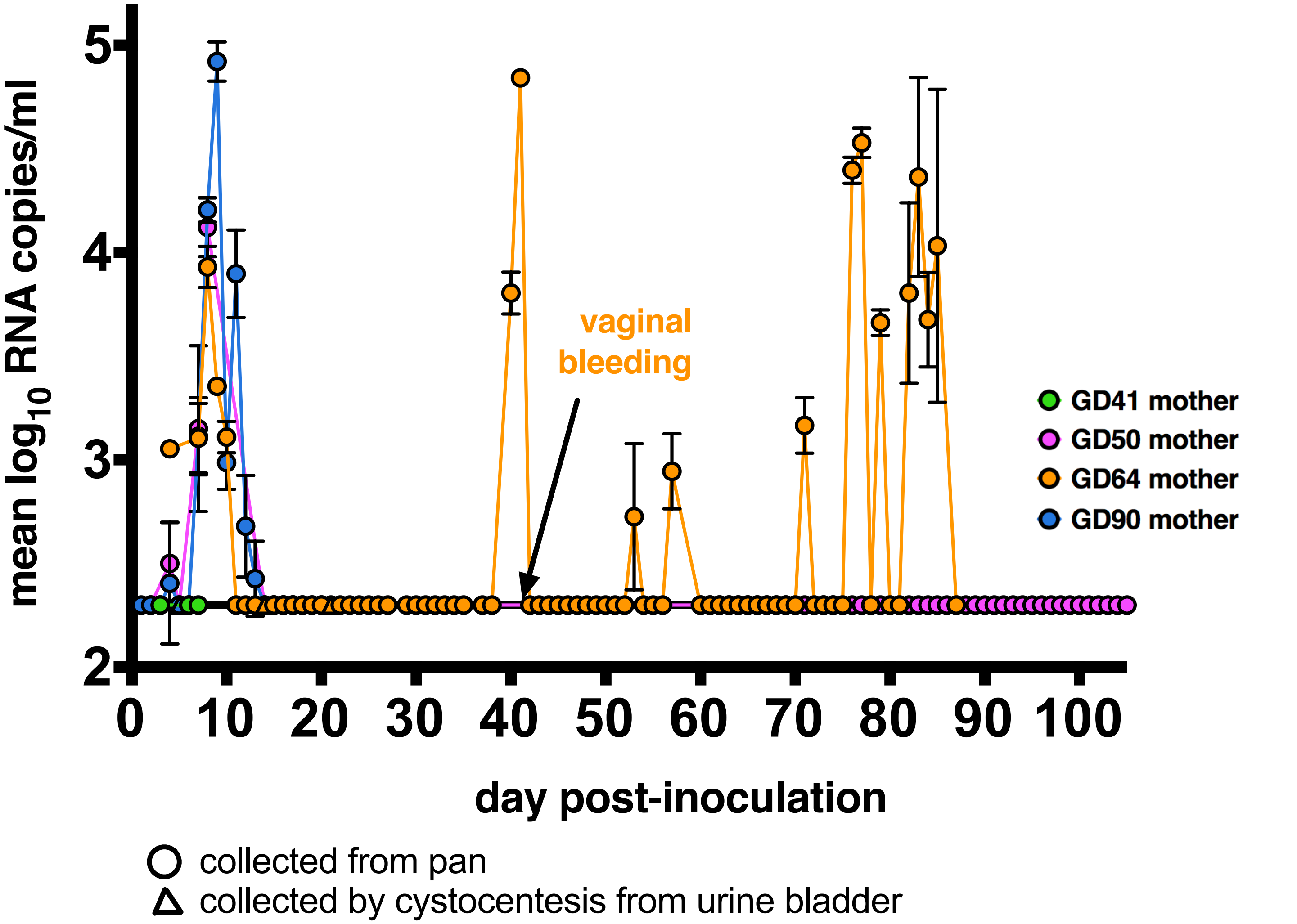

ZIKV RNA levels and kinetics in maternal urine. ZIKV RNA levels in urine collected by cystocentesis from the urine bladder (triangles) or the cage pan (circles), reported in mean log_{10} RNA copies/ml, were assayed in triplicate with standard deviations noted. Each line shows the kinetics for a single animal. The solid line shows the LOD, 2.3 log_{10} RNA copies/ml. The GD64 mother developed placental bleeding with vaginal discharge 39 dpi, which was likely the source of viral RNA in urine collected from the cage pan, as RNA was undetectable in urine collected via cystocentesis. Error bars on RNA measures show standard deviations for 3 replicates.

Supplementary Figure 3

ANTIBODY IN MATERNAL PLASMA

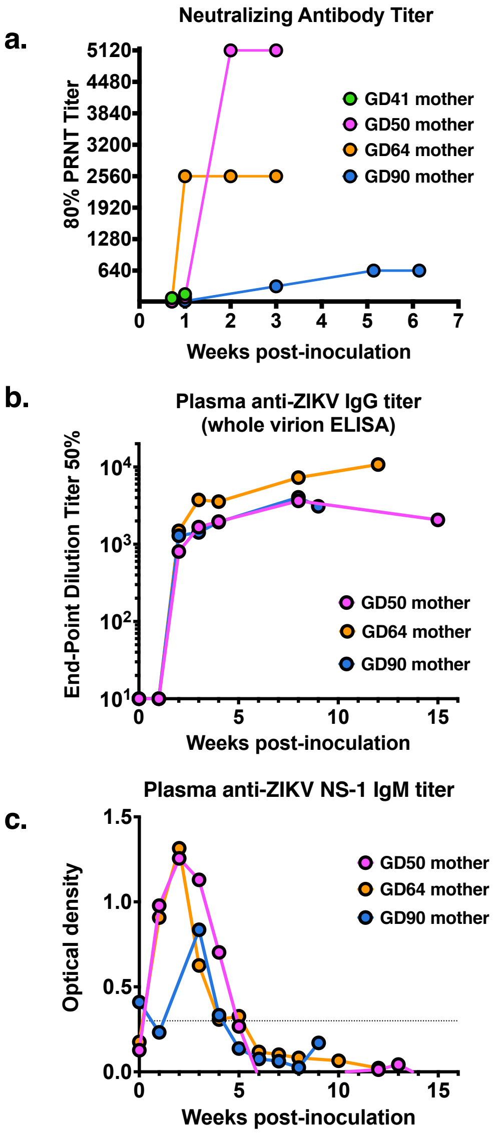

d.

ANTIBODY IN FETUS/NEONATE

	GD50			GD64			GD90		
	IgM	IgG	PRNT	IgM	IgG	PRNT	IgM	IgG	PRNT
**Plasma**	na	na	na	-	+	+	-	-	+
**Amniotic fluid**	-	-	nt	-	+	nt	-	-	nt
**Cerebrospinal Fluid**	-	+	-	-	+	-	-	+	-

Neutralizing and binding antibody kinetics and magnitude in ZIKV-inoculated pregnant macaque plasma and fetal/neonatal fluids. **a)** ZIKV 80% **PRNT**, **b) IgG endpoint titer**, and **c) IgM** OD (by ELISA) in maternal plasma, and **d) IgG, IgM and PRNT reactivity in fetal/neonatal fluids**. The first plasma dilution tested was 1:20. Each line shows the kinetics for a single animal. The GD41 mother was only tested for neutralizing antibody and did not have a detectable response. The dotted line for ZIKV-specific IgM indicates the cut-off value of the ELISA assay. *na* is not available, *nt* is not tested, *CSF* is cerebrospinal fluid. Amniotic fluids tested in d) are from necropsy at 105 dpi for the GD50 animal, 65 dpi for the GD90 animal, and 57 dpi, the last time amniocentesis was performed due to amniotic fluid leakage, in the GD64 animal. CSF samples tested were from necropsy, 105 dpi for the GD50 animal, 87 dpi for the GD64 animal, and 65 dpi for the GD90 animal.

Supplementary Figure 4

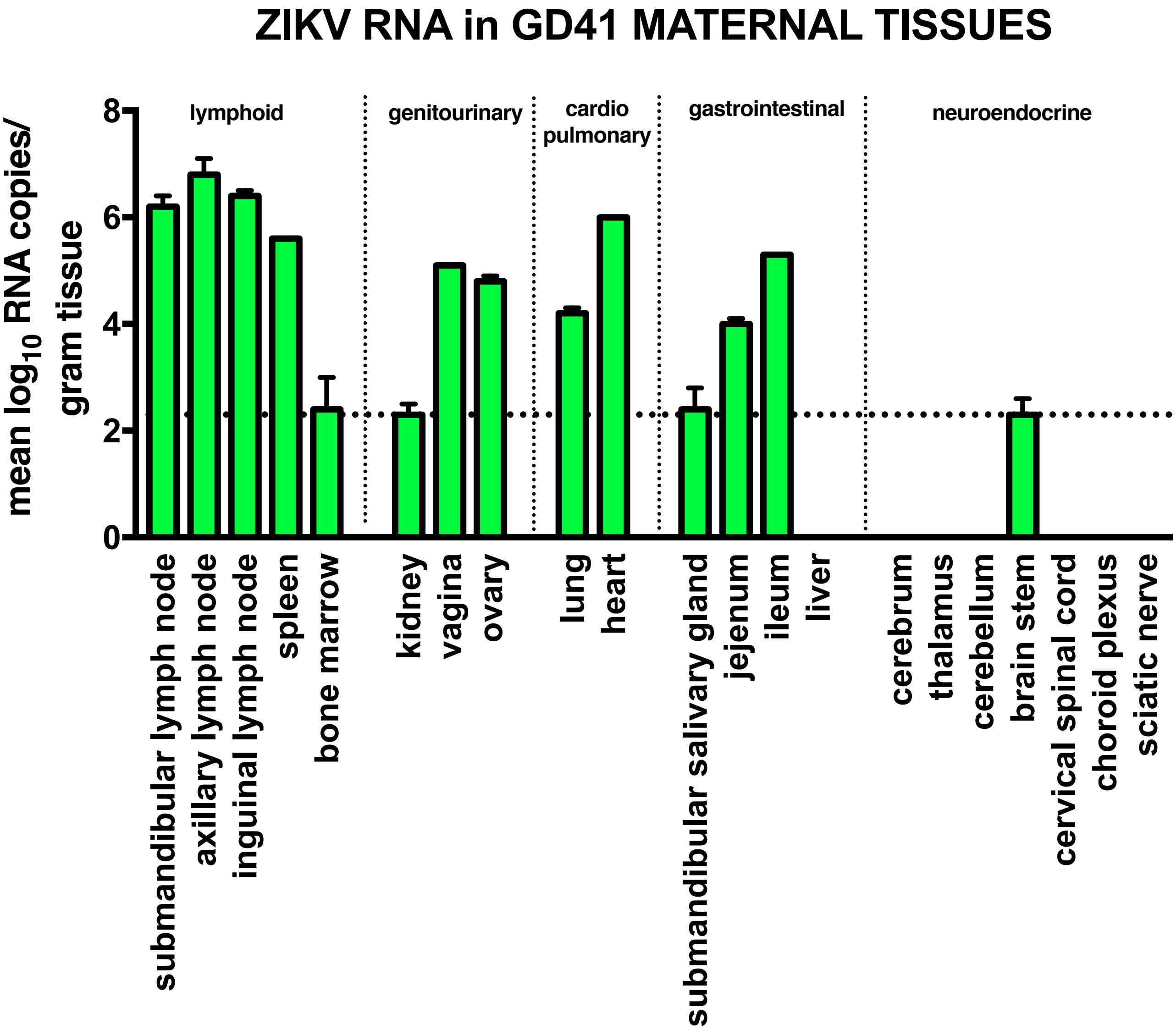

ZIKV RNA levels in GD41 maternal tissues. ZIKV RNA levels represented in mean $\log_{10}$ RNA copies per gram of tissue, assayed in triplicate. The dotted lines show the ZIKV RNA LOD of 2.3 $\log_{10}$ RNA copies. Fewer maternal tissues were collected from this animal compared to the others in the study. Error bars on RNA measures show standard deviations for 3 replicates.

Supplementary Figure 5

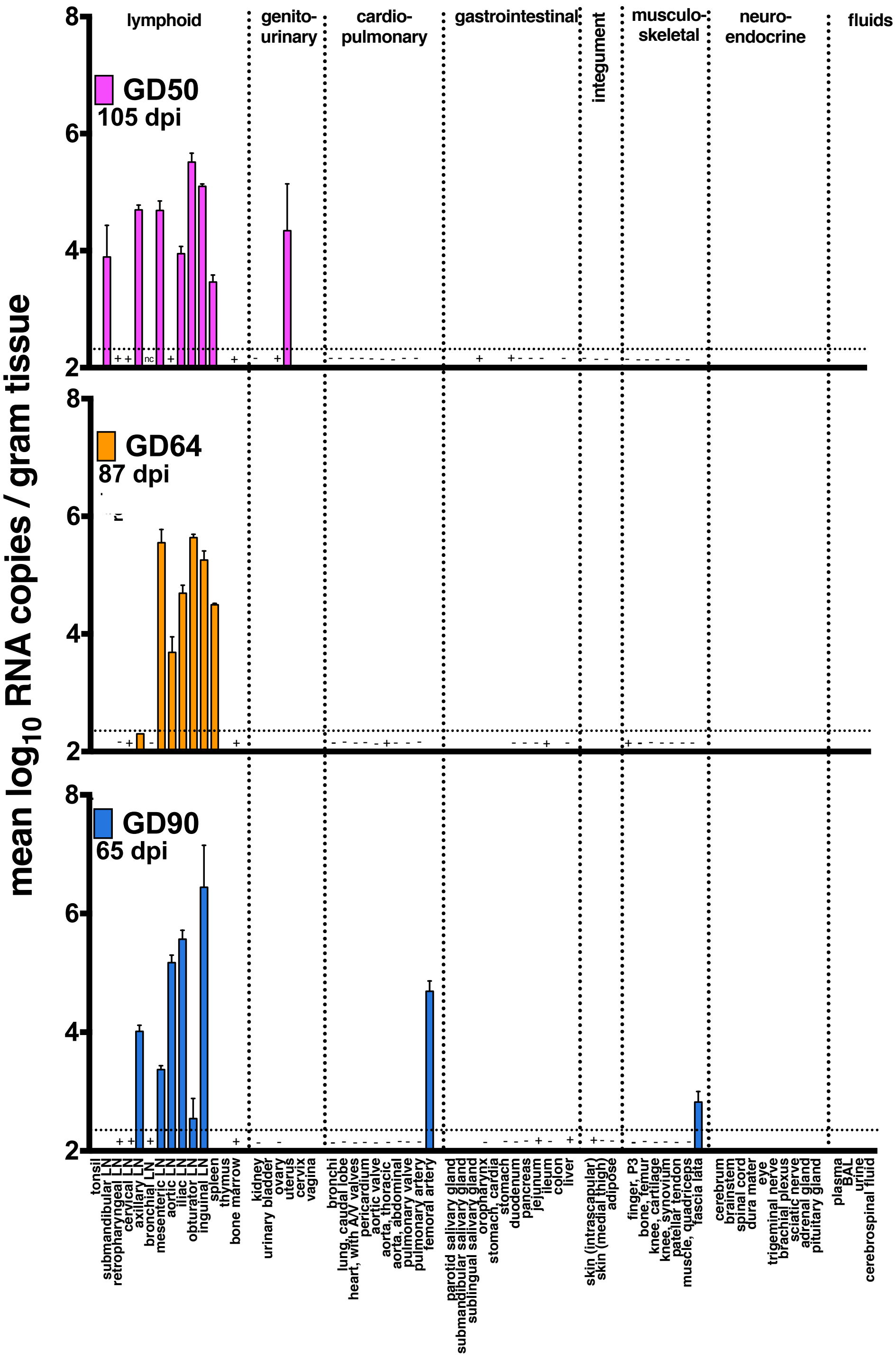

ZIKV RNA in GD50, GD64 and GD90 maternal tissues. ZIKV RNA levels represented in mean $\log_{10}$ RNA copies per gram of tissue, assayed in triplicate. The dotted lines show the ZIKV RNA LOD of 2.3 $\log_{10}$ RNA copies. Bars at the dotted line show samples at the LOD; the absence of bars on tissues indicate samples were tested but no ZIKV RNA signal above the LOD was detected. + symbols above tissue labels indicate samples that showed ZIKV RNA reactivity by the qualitative Procleix assay; the – symbol indicates tissues that were not reactive by Procleix. *nc* is not collected. Error bars on RNA measures show standard deviations for 3 replicates.

Supplementary Figure 6

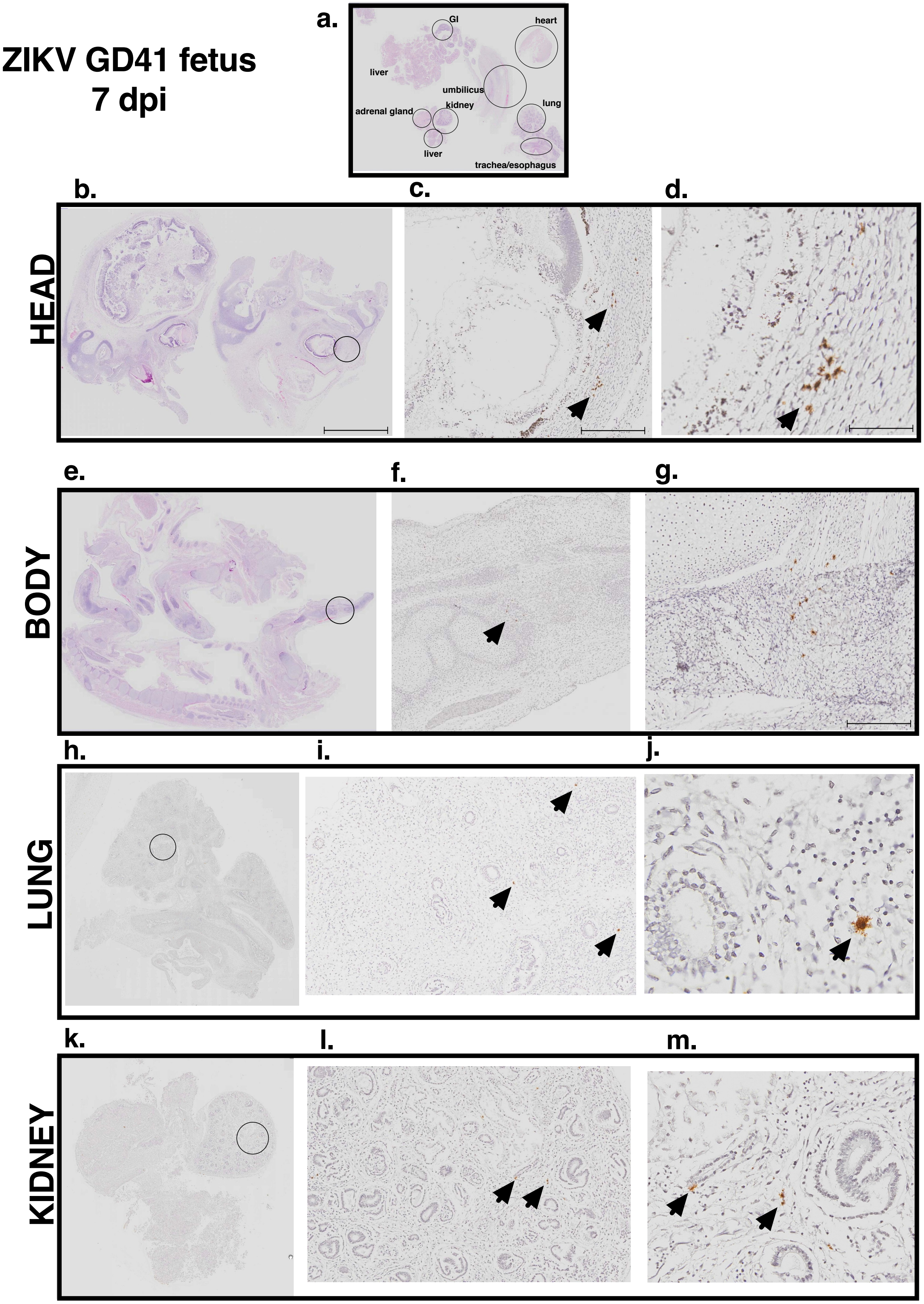

ZIKV RNA *in situ* in GD41 fetal tissues. **a) fetal tissues** and increasing magnifications from left to right of tissue sections of **b-d)** head, **e-g) body, h-j) lung** and **k-m) kidney**. Arrows point to ZIKV RNA labeling by *in situ* hybridization. Scale bars are as follows: b) 400 mm, c) 18 µm, d) 60 µm, g) 95 µm.

Supplementary Figure 7

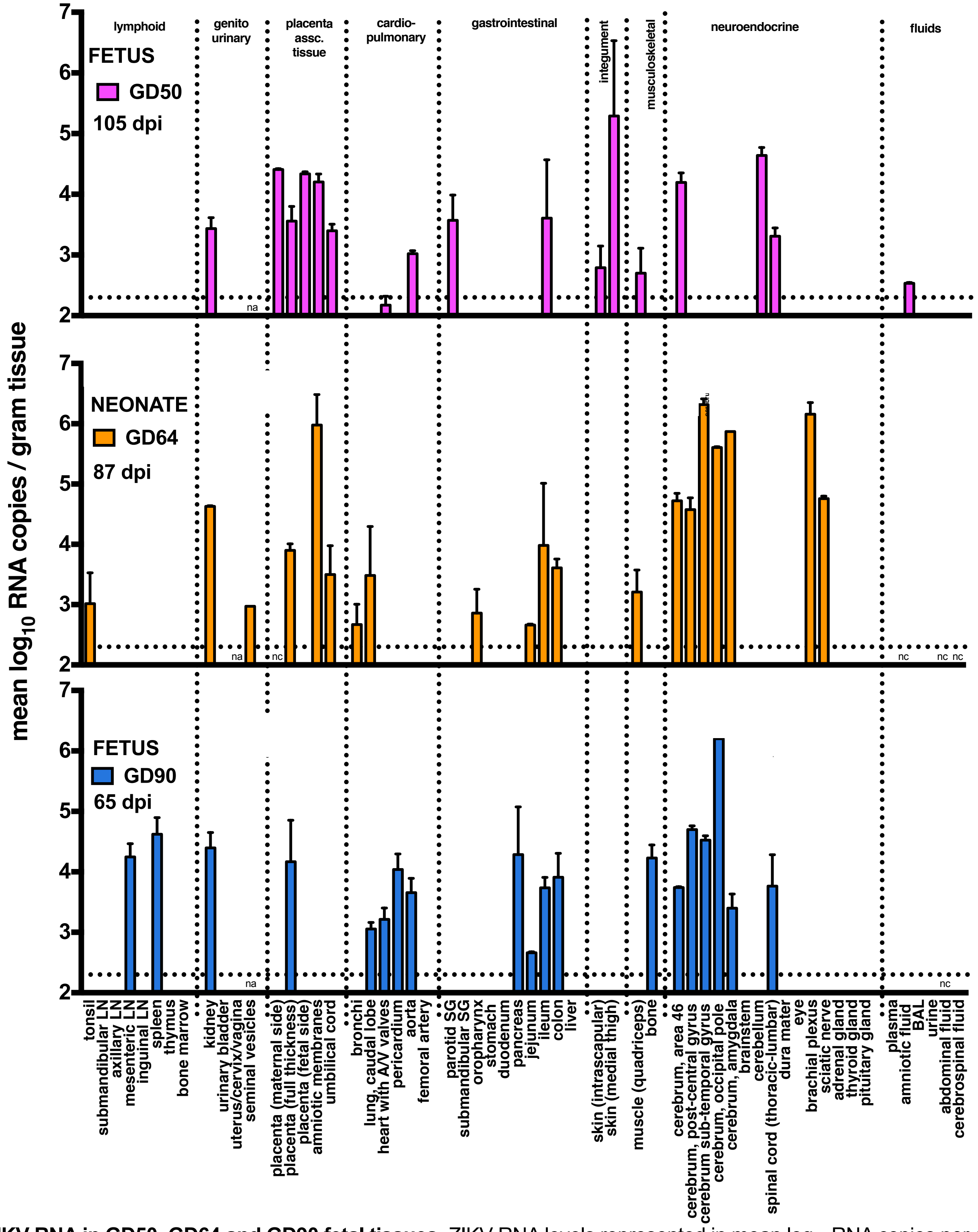

ZIKV RNA in GD50, GD64 and GD90 fetal tissues. ZIKV RNA levels represented in mean log_{10} RNA copies per gram of tissue, assayed in triplicate. The dotted lines show the ZIKV RNA LOD of 2.3 log_{10} RNA copies. Bars at the dotted line show samples at the LOD; the absence of bars on tissues indicate samples were tested but no ZIKV RNA signal above the LOD was detected. Error bars on RNA measures show standard deviations for 3 replicates. Fetal/infant tissues that were not available due to their sex (GD50 and GD90 fetuses were females, GD64 infant was a male) are marked as not applicable (*na*). *LN* is lymph node, *SG* is salivary gland, *MS* is musculoskeletal, *intg* is integument, *BAL* is bronchioalveolar lavage, *A/V* is atrioventricular, *nc* is not collected due absence of sample, which, in the case of the GD64 neonate, was due to live birth.

Supplementary Figure 8

ZIKV GD90 mother

control GD90 mother

a.

b.

c.

d.

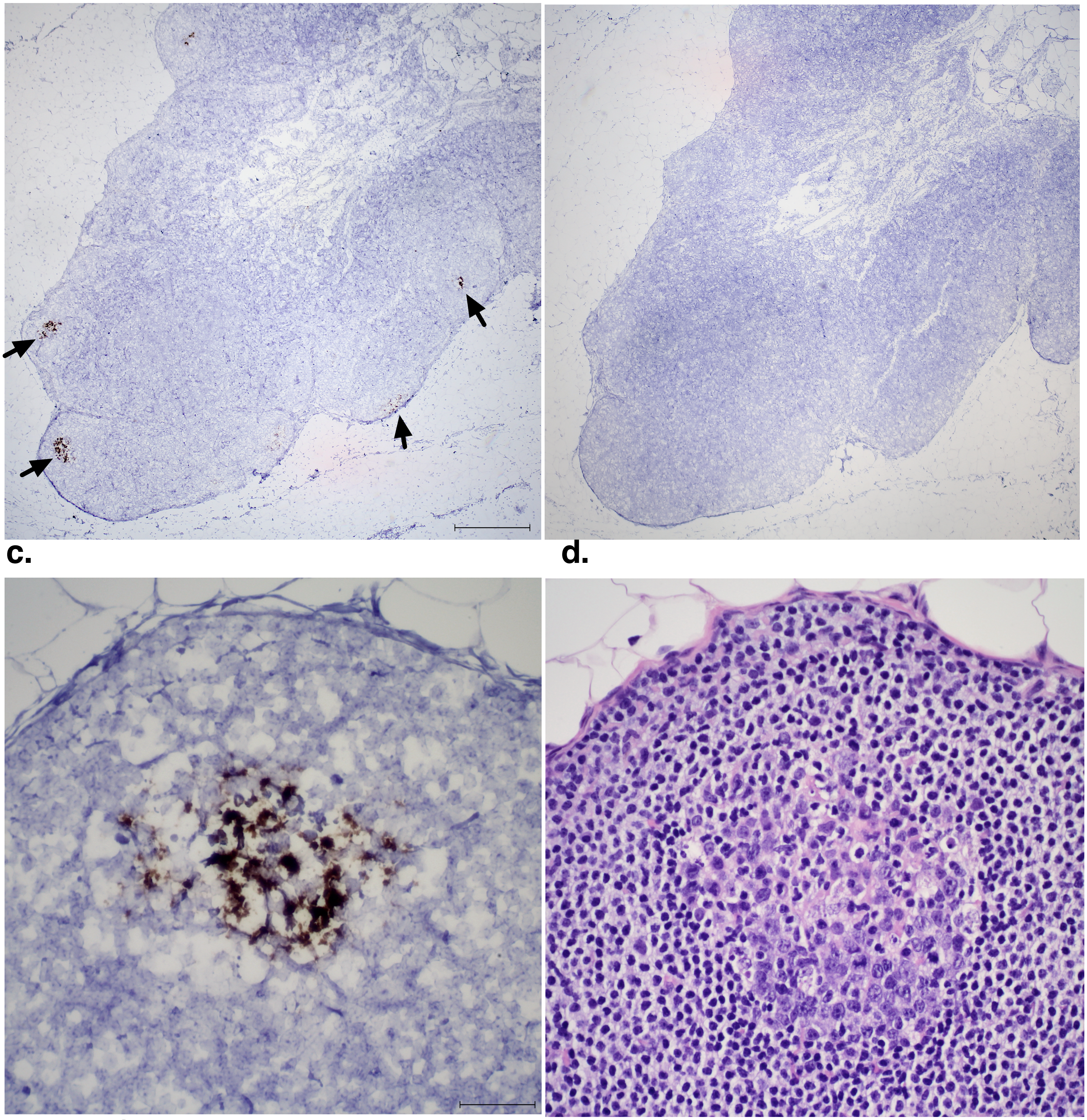

ZIKV GD90 mother

ZIKV GD90 mother

ZIKV RNA *in situ* in GD90 maternal inguinal lymph node. low magnification images of **a)** ZIKV infected GD90 maternal or **b)** control lymph node, **c)** high magnification image of a focus of ZIKV RNA labeling and **d)** H & E in the GD90 ZIKV-infected mother. Arrows point to ZIKV RNA labeling by *in situ* hybridization. Scale bar in a) is 1 mm, c) is 120 µm.

Supplementary Figure 9

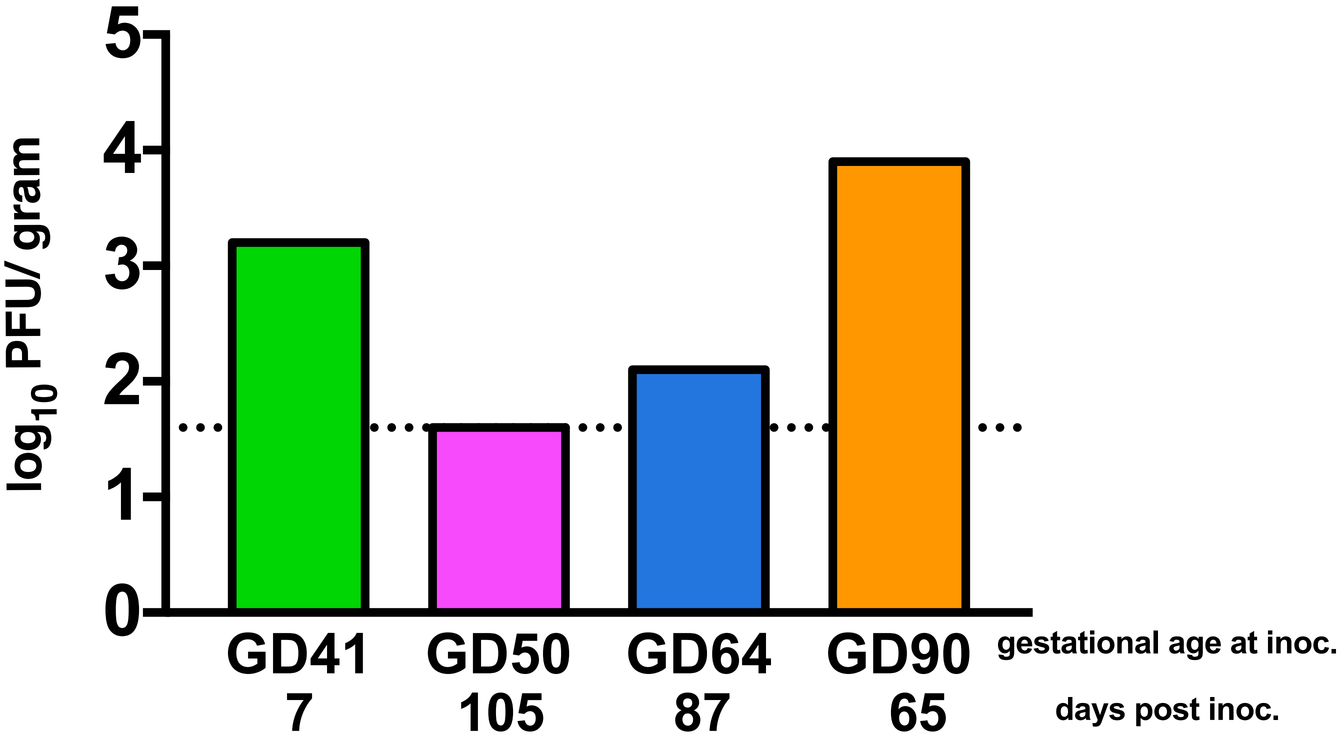

Infectious ZIKV in placentas. Bars show infectious ZIKV at indicated dpi, detected in $\log_{10}$ Vero cell plaque forming units (PFU) per gram tissue. The dotted line shows the LOD of 1.6 $\log_{10}$ PFU per ml.

Supplementary Figure 10

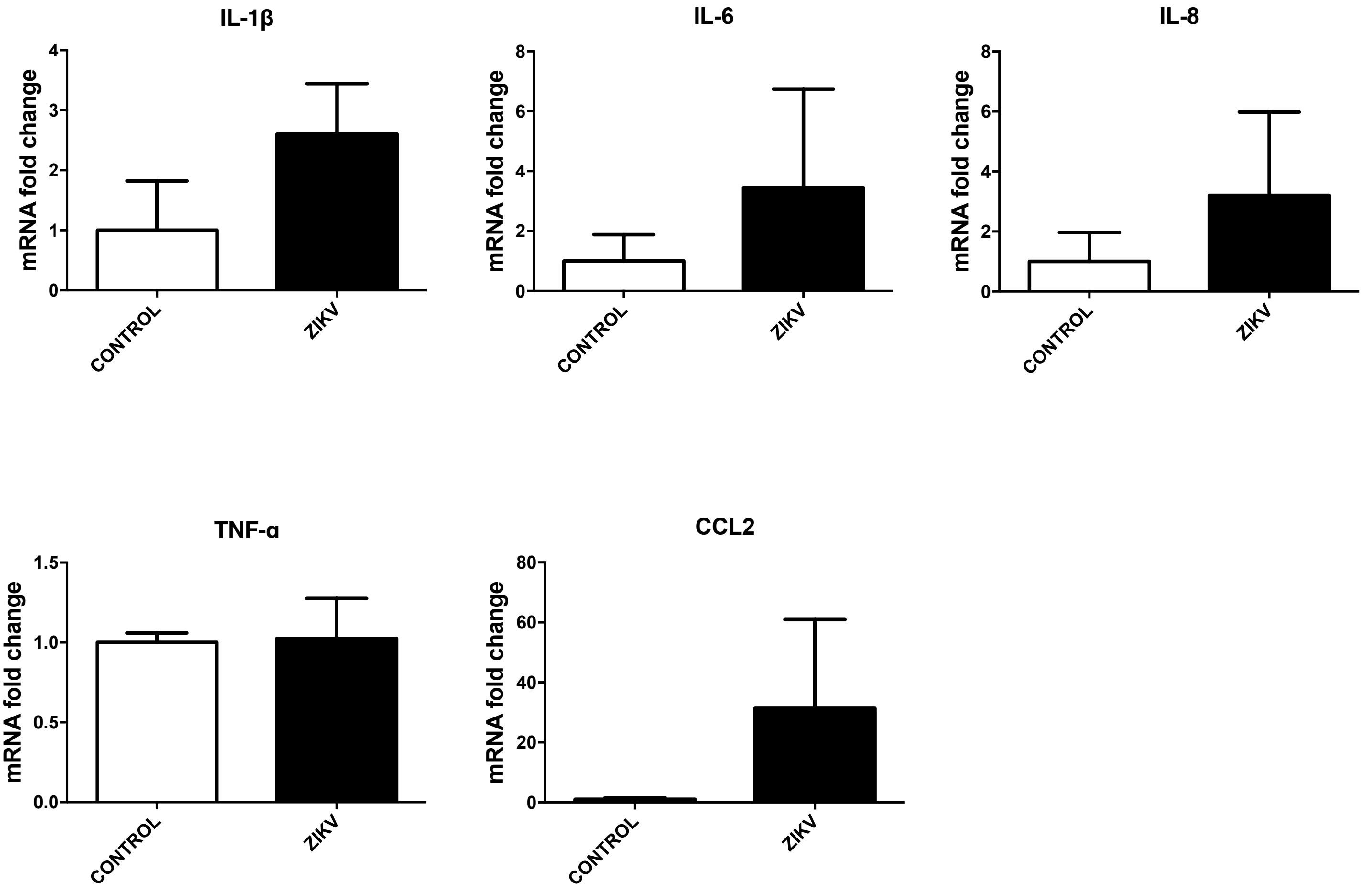

Cytokine mRNAs in chorioamnion decidua from ZIKV infected animals are not different from controls. Proinflammatory cytokine mRNAs were measured in GD-matched control and ZIKV GD50, GD64 and GD90 chorioamnion-decidua tissues (collected at time of delivery or euthanasia) by quantitative RT-PCR. mRNA values were first internally normalized to the endogenous 18S RNA; values reported for each gene in ZIKV animals are expressed as fold increase relative to a pooled average of control animals. Error bars on RNA measures show standard deviations for the group means. None of the differences were statistically significant.

Supplementary Figure 11

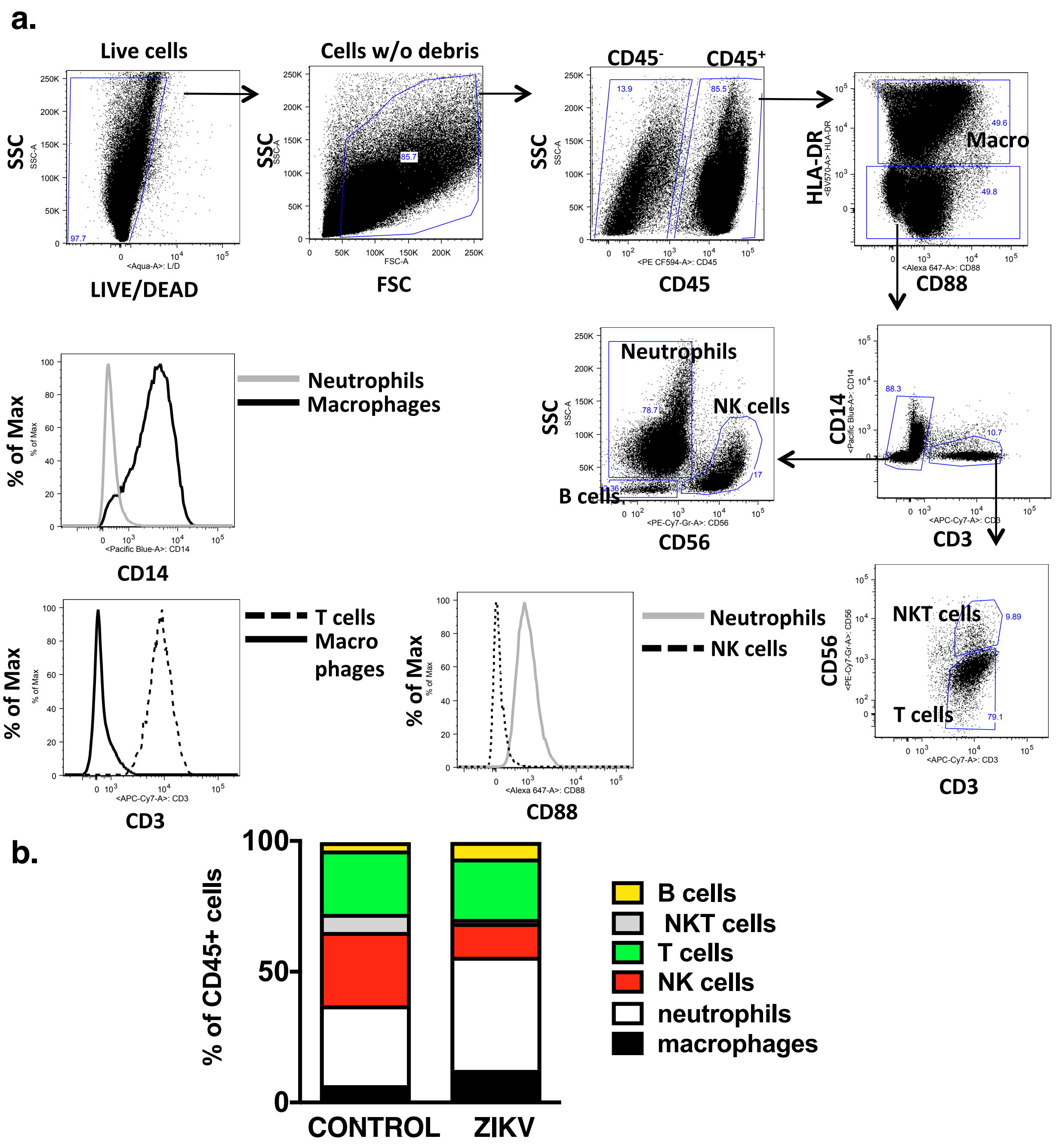

Leukocyte populations in ZIKV GD50, G64 and GD90 rhesus macaque choriodecidua. **a)** A representative gating strategy was used to characterize the different leukocyte subpopulations in the rhesus decidua parietalis of ZIKV infected macaques. Live cells were first identified by the absence of LIVE/DEAD stain and forward-/side-scatter expression, excluding cell debris. Then, leukocytes were gated as $CD45^+$ cells. Inside the $CD45^+$ cells, the leukocyte subpopulations were gated as monocytes/macrophages ($CD3^-CD14^{high}CD88^+HLA\text{-}DR^+$); neutrophils ($CD3^-CD14^{low}HLADR^-CD88^+CD56^-$); NK cells ($CD3^-CD14^-HLA\text{-}DR^-CD88^-CD56^+$); B cells ($CD3^-CD14^-CD56^-CD19/CD20^+$); T cells ($CD14^-CD56^-CD3^+$); and NKT cells ($CD14^-CD3^+CD56^+$). **b)** Chorio-decidua cells were scraped, digested with protease/DNAase and single cell suspensions were used for multiparameter flow cytometry phenotyping of different leukocyte populations. *NK* is natural killer.

Supplementary Figure 12

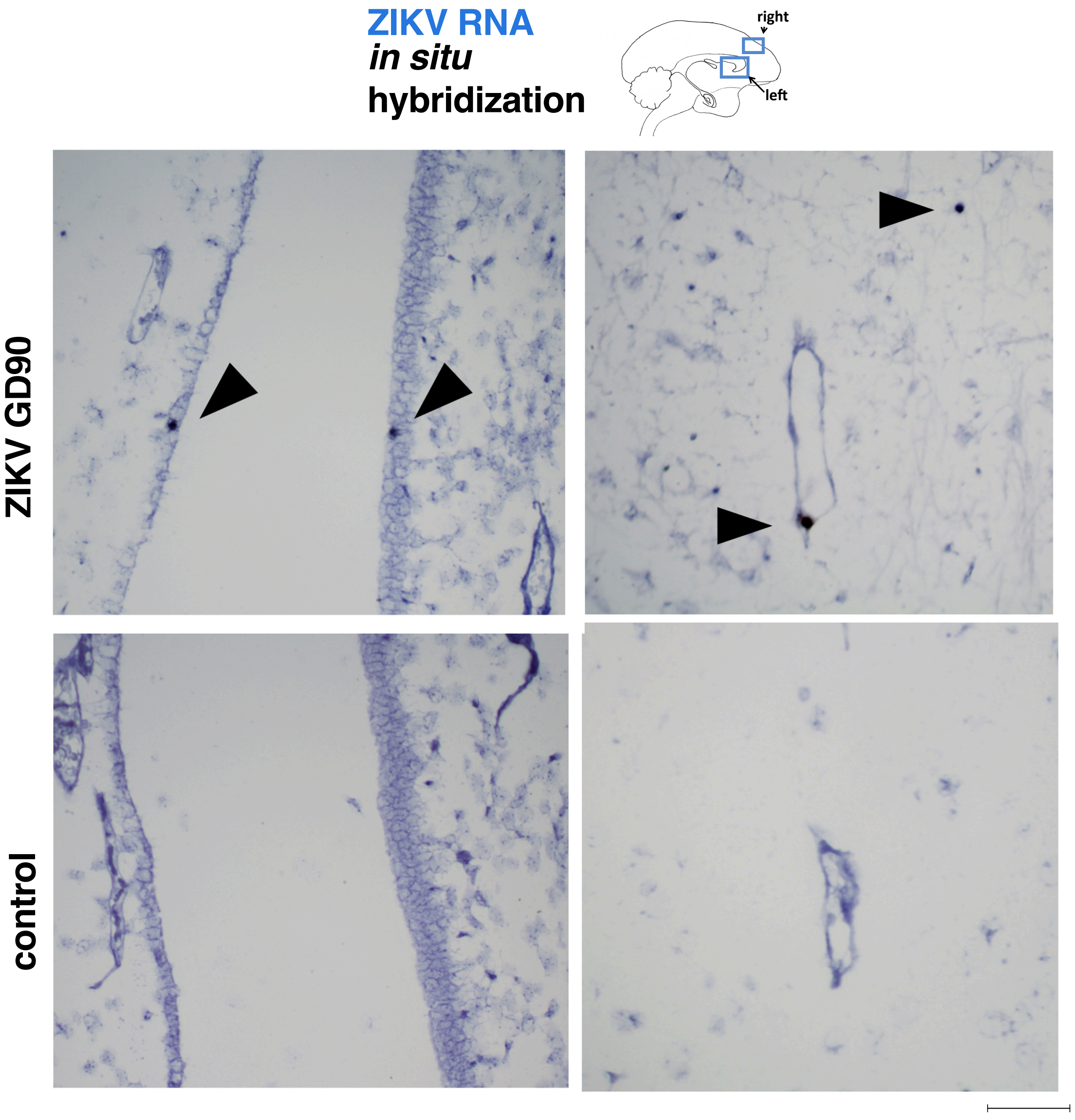

ZIKV RNA is detectable in fetal brain tissues. *In situ* hybridization staining (brown dots, highlighted by arrows) showing ZIKV RNA in GD90 fetal brain (upper) with scattered neurons in the neuropil adjacent to a vessel in the cerebral cortex (left) and ependymal lining of the lateral ventricle (right) compared with GD90 control fetal brain (lower) showing no ZIKV RNA labeling. Scale bar shows 120 µm. Brain sketch shows regions imaged.

Supplementary Figure 13

NEURAL PROGENITOR CELL MARKER IHC
subventricular
Sox2

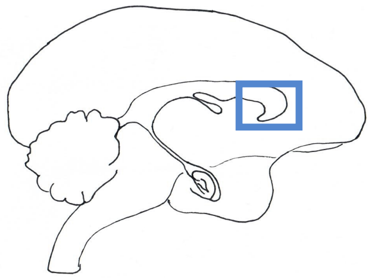

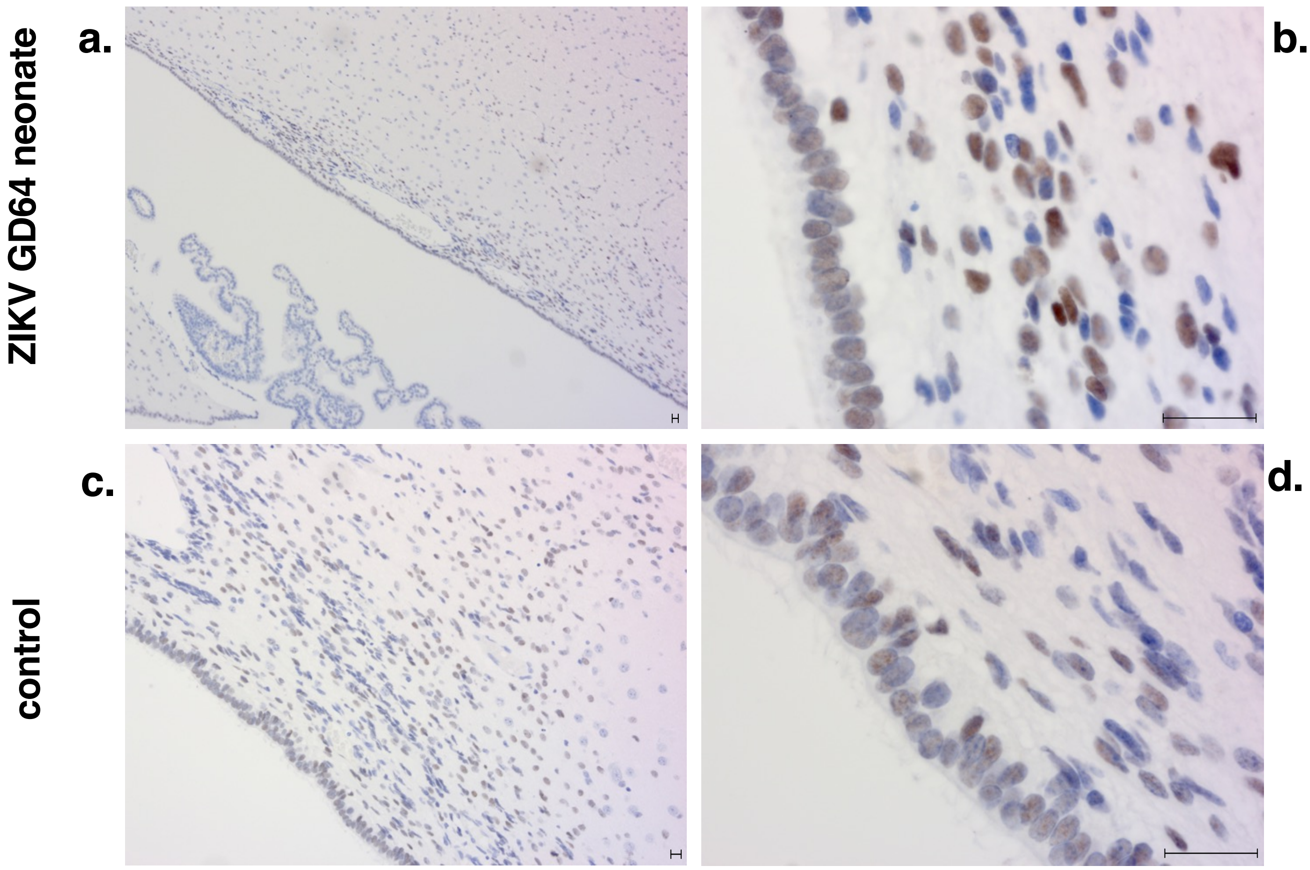

Neural stem cell markers in subventricular zone of ZIKV infected animals are unchanged compared to controls. Immunohistochemistry (brown staining) labels Sox2 in the subventricular zone at low (40X) and high (400X) magnifications at similar levels in the **a-b)** ZIKV GD64 neonate and **c-d)** control. Scale bars show 20 µm. Brain sketch shows regions imaged.

Supplementary Figure 14

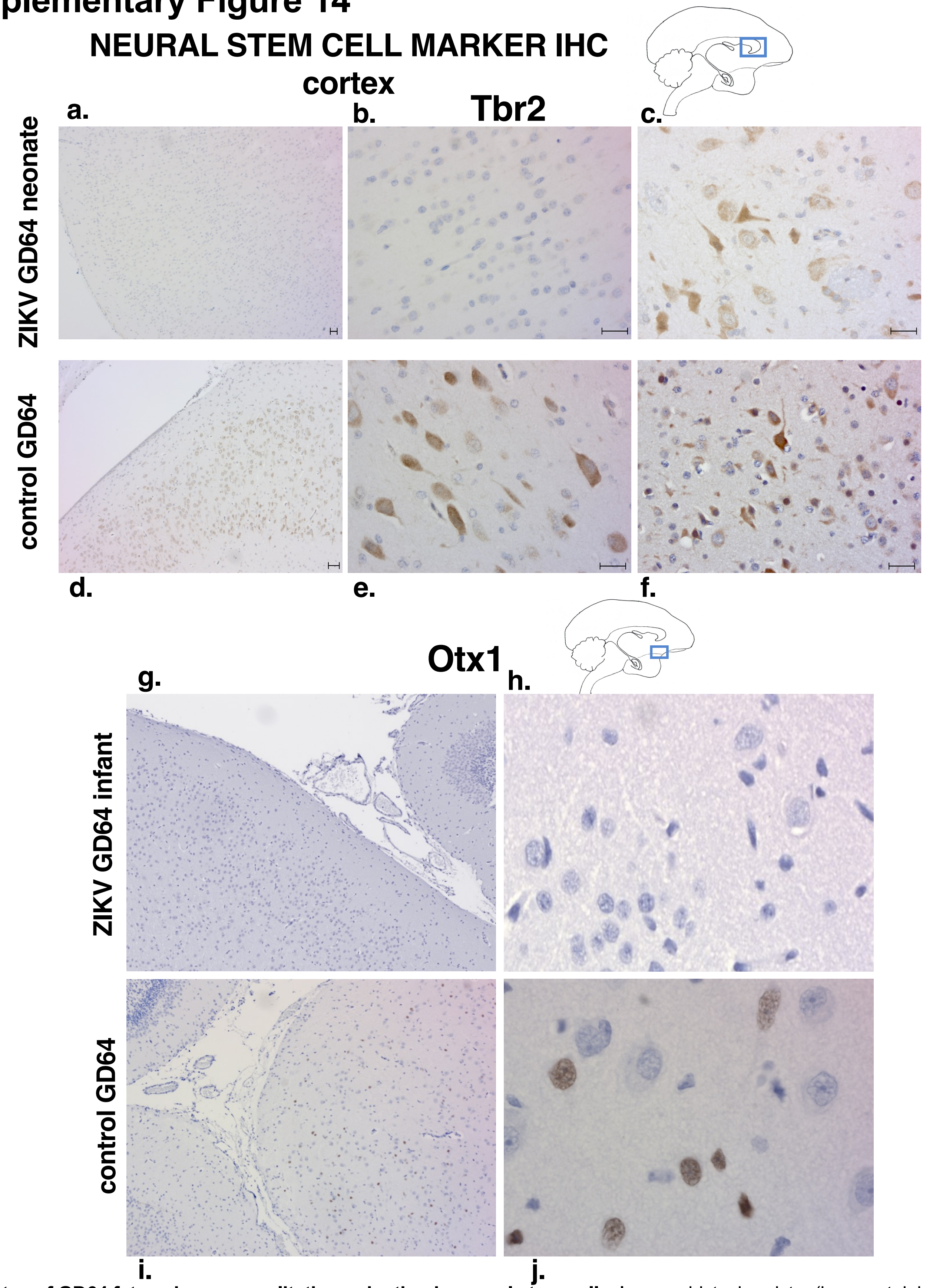

Cortex of GD64 fetus shows a qualitative reduction in neural stem cells. Immunohistochemistry (brown staining) labels Tbr2 in the **a-c)** ZIKV GD64 neonate versus **d-f)** control animal, 40X left, 400X middle and right) and Otx1 in the **g-h)** ZIKV GD64 neonate versus **i-j)** control animal, magnification 40X left, 400X right. Scale bars show 20 µm. Brain sketches show regions imaged.
